# iSheep: an Integrated Resource for Sheep Genome, Variant and Phenotype

**DOI:** 10.3389/fgene.2021.714852

**Published:** 2021-08-17

**Authors:** Zhong-Huang Wang, Qiang-Hui Zhu, Xin Li, Jun-Wei Zhu, Dong-Mei Tian, Si-Si Zhang, Hai-Long Kang, Cui-Ping Li, Li-Li Dong, Wen-Ming Zhao, Meng-Hua Li

**Affiliations:** ^1^National Genomics Data Center, Beijing Institute of Genomics, Chinese Academy of Sciences (China National Center for Bioinformation), Beijing, China; ^2^College of Life Sciences, University of Chinese Academy of Sciences (UCAS), Beijing, China; ^3^CAS Key Laboratory of Animal Ecology and Conservation Biology, Institute of Zoology, Chinese Academy of Sciences, Beijing, China; ^4^College of Animal Science and Technology, China Agricultural University, Beijing, China

**Keywords:** iSheep, databases, variant, phenotype, annotation

## Introduction

Sheep (*Ovis aries*), one of the main and oldest livestock in the world, are particularly beneficial to human society by supplying wool, meat, milk, and skins. They have been domesticated *ca*. 8,000–12,000 years B.P. (Zeder, 2008). Being one of the earliest domesticated animals and one of the closest animals to human, sheep are also useful in revealing the history of early human settlements and expansions by analyzing their patterns of genetic variants (Zhao et al., 2017; Hu et al., 2019a; Deng et al., 2020).

The completion of a sheep reference genome (Jiang et al., 2014) and rapid advancement in high-throughput sequencing technologies have greatly accelerated the understanding of domestication, evolution and genetic mechanisms underlying various phenotypic traits in sheep (Lv et al., 2014; Yang et al., 2016; Alberto et al., 2018; Naval-Sanchez et al., 2018; Li et al., 2020). With the amount of increasing genomic data, establishing a systematic database in sheep for data archiving, analyzing and visualization becomes particularly essential, since so far only few databases are available for sheep compared with a variety of integrated resources established in mice (Laulederkind et al., 2013), dogs (Tang et al., 2019) and cattle (Elsik et al., 2016).

To date, one of the most widely accessible genetic databases for sheep is the International Sheep Genomics Consortium (ISGC, https://www.sheephapmap.org/). The ISGC database contains sheep genome assemblies and variants of 935 sheep representing 69 breeds from 21 countries. The ISGC database consists of around 50 million filtered variants called using GATK and Samtools programs based on the reference genome assembly Oar_v3.1. Also, it comprises the results at European Variation Archive (EVA) and the genotypes of several SNP chip arrays (Illumina 15K, 50K and HD 600K SNP chips). For the EVA database, users can't obtain complete VCF files in its variant browser. Meanwhile, the EVA data set has gathered a large amount of genetic data, but the information on raw sequencing, annotation, breed and phenotype still remains underdeveloped. Another public database, dbSNP (https://www.ncbi.nlm.nih.gov/snp/), was established in 1999 by the National Center for Biotechnology Information (NCBI, https://www.ncbi.nlm.nih.gov/), which has collected variation information of *Homo sapiens, Mus musculus* and other species (Sherry et al., 2001). However, dbSNP has discontinued to update non-human variations since 2017 (https://ncbiinsights.ncbi.nlm.nih.gov/2017/05/09/phasing-out-support-for-non-human-genome-organism-data-in-dbsnp-and-dbvar/#more-1122), which brings inconvenience for research communities to use non-human variations. As a complementary resource to the dbSNP, the Genome Variation Map (GVM, https://ngdc.cncb.ac.cn/gvm/) is dedicated to collecting, integrating, and visualizing different types of genome variations for a series of species from all over the world (Song et al., 2018; Li et al., 2021). Up to 2021, the latest version of GVM has included 355 next generation sequencing (NGS) deposits of wild and domestic sheep (Li et al., 2021), which is a large data set, but lacks functional genetic variants and related information.

Here, we present iSheep (https://ngdc.cncb.ac.cn/isheep/), a specialized, integrated, and open-access resource for sheep. It consists of whole genome raw sequencing data, genomic variants, functional annotations, breed information and phenotypic traits (including morphological, production and disease-resistance traits). It also provides a world-wide public and free data service. Furthermore, iSheep incorporates online data analysis tools for data mining, genome navigation and annotation, which will not only be useful for the sheep research communities but also benefit a large number of sheep breeders.

## Materials and Methods

**Figure 1:**
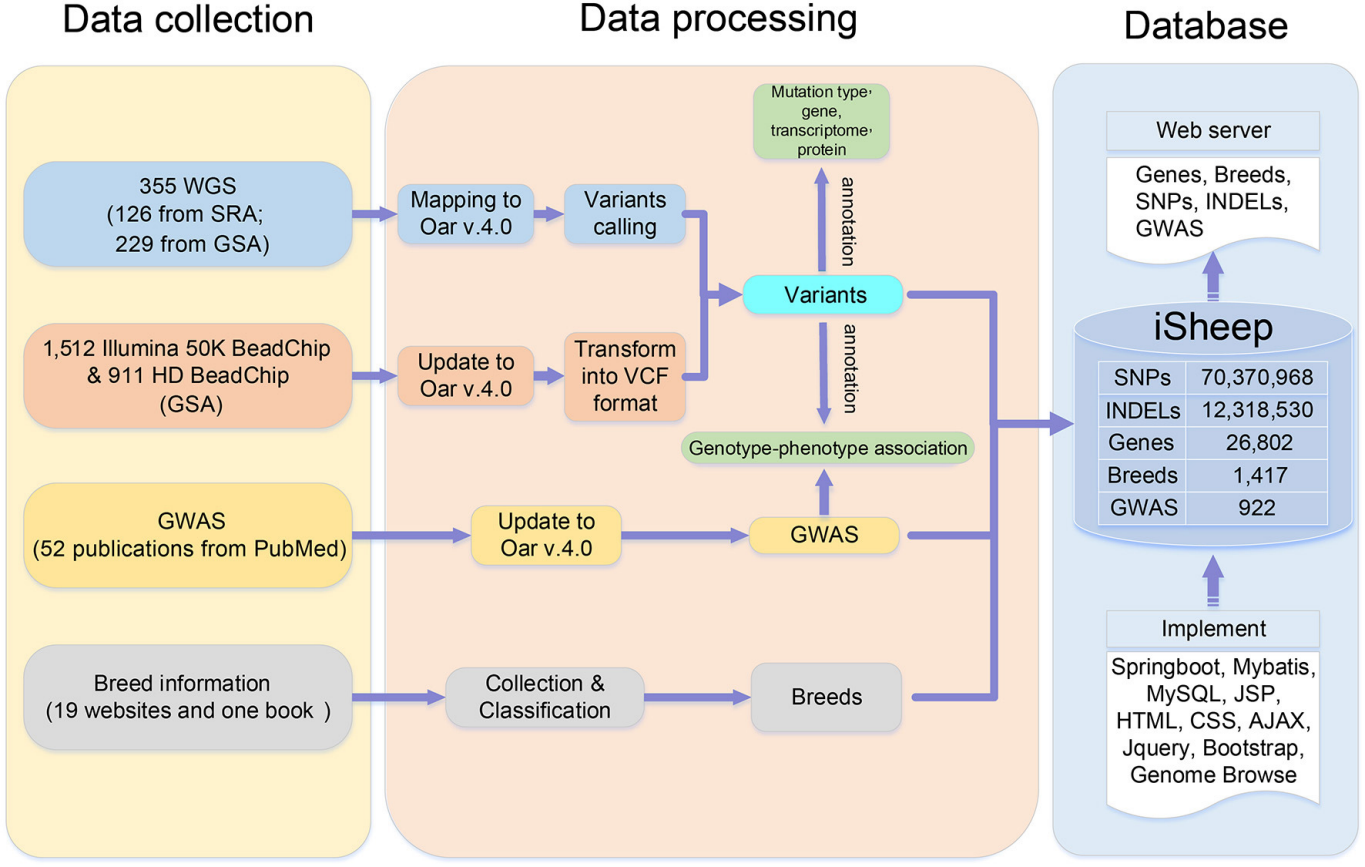
Data sources and pipelines of iSheep.

The pipeline for database construction is shown in Figure 1, and details are described in Data collection, Data processing, and Database implementation.

### Data Collection

Whole-genome sequencing (WGS) data with a depth of 4~40 × coverage were collected from two sources: 126 samples from the GVM database, and 229 samples from Yang et al. (2016) and Hu et al. (2019a). All the raw WGS data were deposited in the Genome Sequence Archive (GSA, https://ngdc.cncb.ac.cn/gsa/), which is a core data resource of the National Genomics Data Center (NGDC, https://ngdc.cncb.ac.cn/) and archives raw sequence data.

The Ovine SNP BeadChip data including Ovine Illumina 50K BeadChip of 1,512 samples and Ovine Infinium HD 600K SNP BeadChip of 911 samples are from seven published papers (Aken et al., 2016; Ren et al., 2016; Peng et al., 2017; Xu et al., 2017, 2018; Chen et al., 2018; Gao et al., 2018) (Supplementary Table 1). All the Illumina 50K BeadChip and HD 600K BeadChip data were merged and submitted separately to the GVM.

The breed information was derived from 19 public websites and a book (Supplementary Table 2). The information on GWAS was acquired from 52 published papers using genome-wide association studies (GWAS) method in PubMed (Supplementary Table 3).

### Data Processing

Whole genome sequence (WGS) reads were mapped to the sheep reference genome Oar v4.0 (https://www.ncbi.nlm.nih.gov/assembly/GCF_000298735.2) by Burrows-Wheeler Aligner (BWA) v.0.7.10-r789 (Li and Durbin, 2009). Mapping results were then converted into BAM format and sorted by SortSam in Picard package v.2.1.1 (“Picard Toolkit.” 2019. Broad Institute, GitHub Repository. http://broadinstitute.github.io/picard/; Broad Institute). MarkDuplicates in Picard was used to remove duplicated reads. INDEL realignment and correction of base quality were performed through GATK v.3.7 (McKenna et al., 2010). HaplotypeCaller and GenotypeGVCFs in GATK were then used for variants calling and joint genotyping. After filtering, the non-redundant variants were identified and assigned with ‘oas' number corresponding to ‘rs' number in European Variation Archive (EVA) (https://www.ebi.ac.uk/eva/). SNP Chip data of Illumina 50K BeadChip and HD BeadChip were updated to Oar v.4.0, transformed into VCF format using plink v.1.9 (Purcell et al., 2007), and then mapped to the WGS variant sites identified above.

We integrated the variants obtained above according to the position on the chromosomes, performed annotations of variants using VEP v.84 (McLaren et al., 2016), and obtained corresponding information on genes, transcriptomes, and proteins. We also calculated minor allele frequency (MAF) for each variant using vcftools v.0.1.13 (Danecek et al., 2011). Besides, NCBI Genome Remapping Service (https://www.ncbi.nlm.nih.gov/genome/tools/remap) was also used to find corresponding variant position in Oar Rambouillet v.1.0 from Oar v.4.0.

Breed information was collected from the public repositories, including Wikipedia, Domestic Animal Diversity Information System (DAD-IS 3), Breeds of Livestock, Sheep101, Roy's Farm, and Animal Genetic Resources in China: Sheep and Goats (Supplementary Table 2). To provide a unified description of phenotype, we defined a few rules to standardize the breed information, for example, using the most popular name to nominate the breed name. GWAS information was curated from published papers associated with GWAS in sheep manually. Among 152 related publications, 922 Associations relative to 110 traits were extracted from 52 papers published from the years of 2011 to 2019 (Supplementary Table 3). Finally, we used the online tool LiftOver (http://genome.ucsc.edu/cgi-bin/hgLiftOver) to correct the coordinates of each curated genotype.

### Database Implementation

iSheep is implemented by frameworks of springboot version 1.5.9 (https://spring.io/projects/spring-boot/) and mybatis version 1.3.1 (https://mybatis.org/), and data were stored and retrieved through MySQL 8.0 (http://www.mysql.org; a free and popular relational database management system). Web user interfaces were developed by JSP (JavaServer Pages; a technology facilitating rapid development of dynamic web pages based on the Java programming language), HTML (HyperText Markup Language), CSS (Cascading Style Sheets), AJAX (Asynchronous JavaScript and XML; a set of web development techniques to create asynchronous applications without interfering with the display and behavior of the existing page), JQuery (a cross-platform and feature-rich JavaScript library; http://jquery.com, version 3.3.1) and Bootstrap (https://getbootstrap.com, version 4.1.3). Genomic visualization was achieved by Dalliance (Down et al., 2011).

## Results

### Data Contents and Statistics

**Figure 2:**
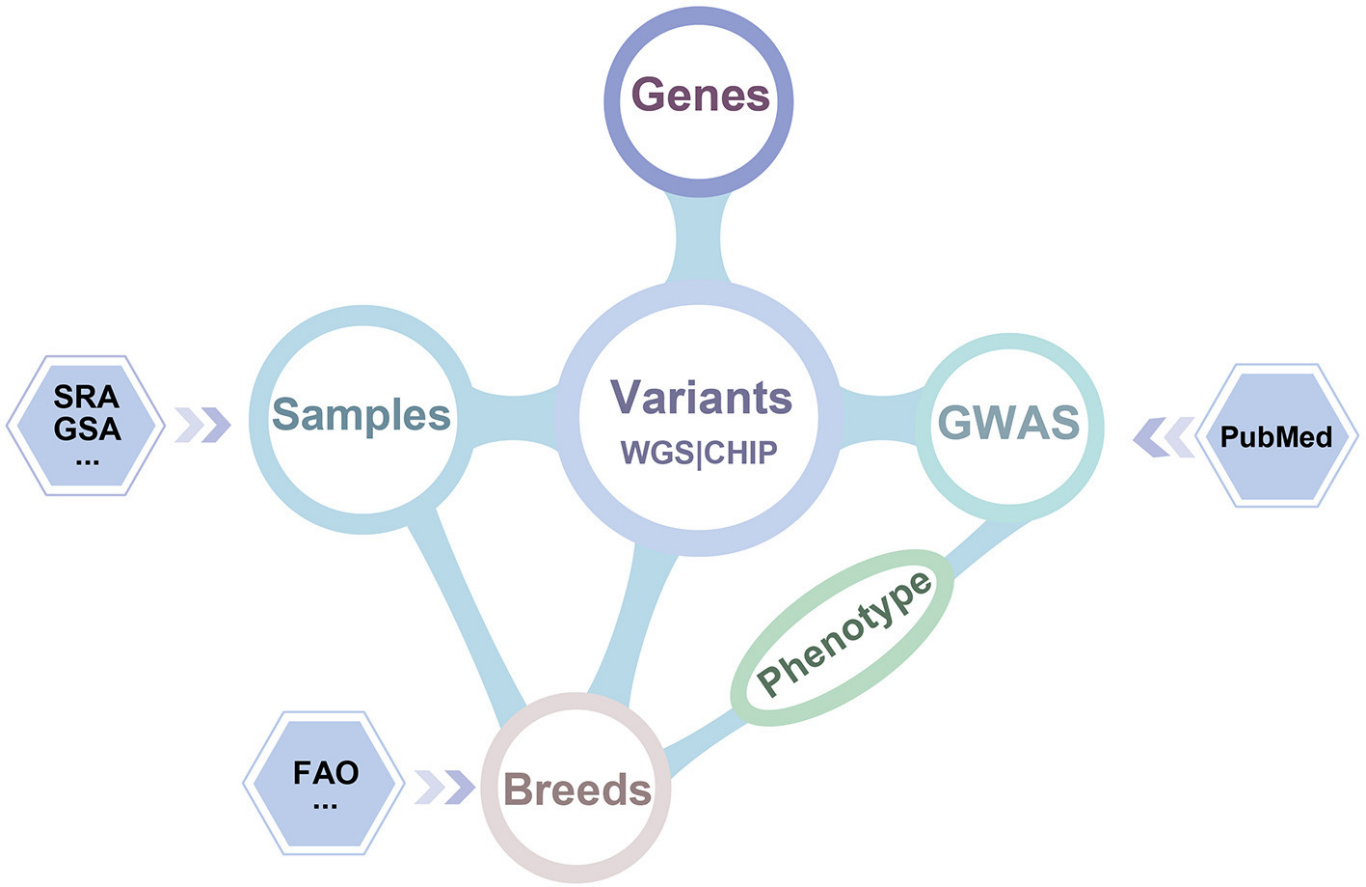
Dataset relationship of iSheep. The iSheep integrates multiple types of data set with inter-connections between each other: (*i*) The Variants called from 355 samples' whole genome sequence data and 2,423 samples' chip data which have been deposited in Genome Sequence Archive (GSA) and Sequence Read Archive (SRA); (*ii*) Genes with variation annotation; (*iii*) GWAS dataset containing manually curated publications of genome-wide variant-trait associations in specific breeds; (*iv*) The sample information and corresponding breed information.

iSheep integrates phenotypic and genotypic data modules containing Breeds, Samples, Genome-wide association study (GWAS), Variants and Genes. The modules (i.e., Breeds, Samples, GWAS and Genes) can be related by the module Variants (Figure 2). In details, we collected whole-genome sequencing (WGS) data of 355 sheep, SNP BeadChip data of 2,423 sheep, 26,802 genes annotated in the sheep genome, 1,417 breeds, and 922 variant-trait associations from 52 publications. Moreover, different data sets have been translated into usable information through standard data processing (Figure 1). Also, we provide a unified data service for the sheep research communities.

**Figure 3:**
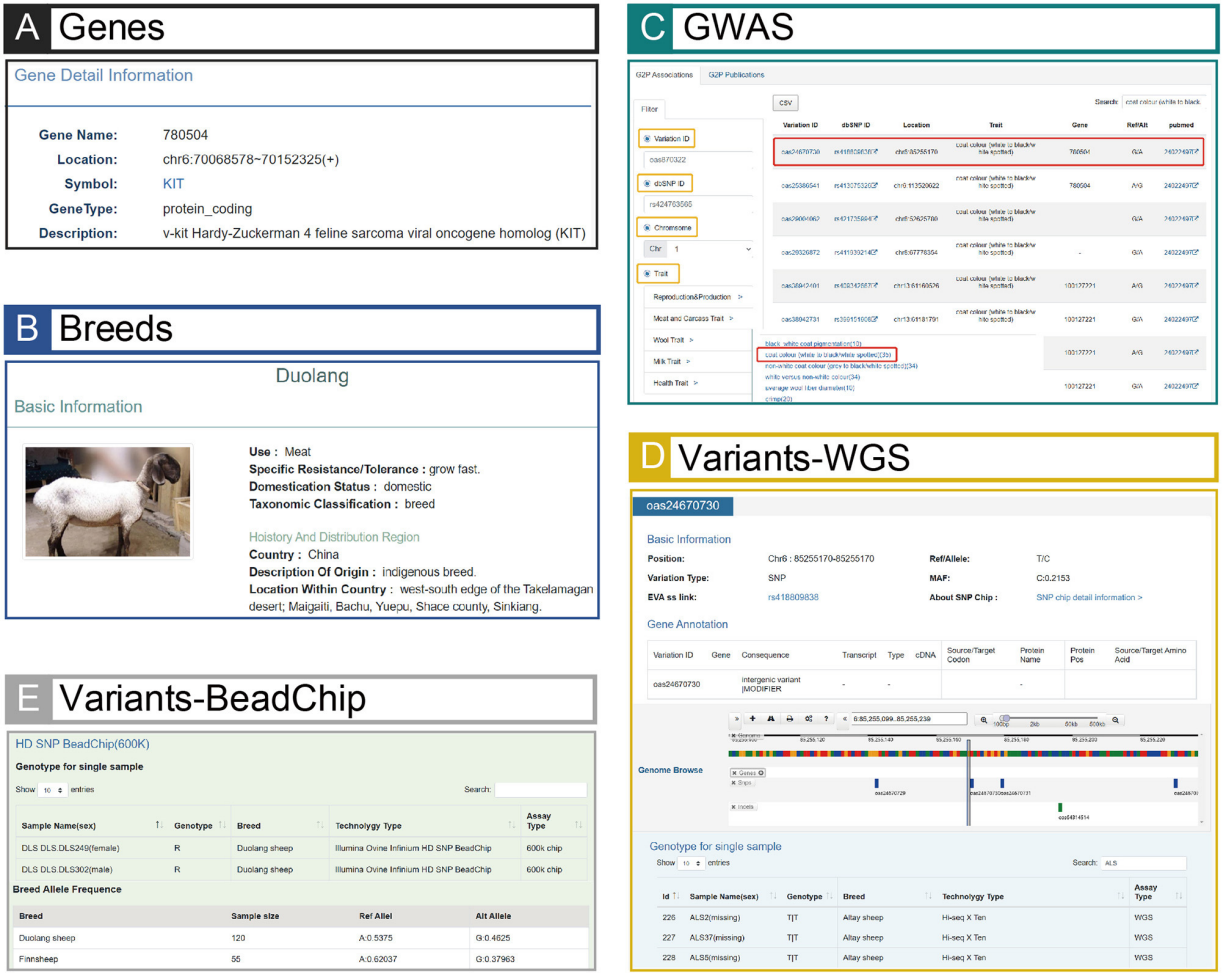
Screenshots of GWAS, Variants, Breeds and Genes modules. **(A)** An example searching result of GWAS. The result page is linked to Genes **(E)** and Variants **(B)**, and the page can jump to corresponding pages when relevant buttons (e.g., variant ID: oas24670730, or gene name: 780504) are clicked. **(B)** An example result of Variants (variant ID: oas24670730). The result page shows detailed information of this example and links to extensional information of BeadChip data **(C)**. **(C)** An example result in BeadChip of Variants (variant ID: oas24670730). The result page is linked to **(B)** when the users click “SNP chip detail information >”, and Duolang sheep with the highest mutation frequency in this example is shown in the table. **(D)** Phenotypic data of Duolang sheep. **(E)** Page of the gene (gene name: 780504) related to the variant (variant ID: oas24670730) in **(A)**.

In total, the information on variants contains 70,370,968 SNPs and 12,318,530 INDELs (Table 1), and it was annotated to 24 consequence types. The results showed that the variants located in noncoding regions (e.g., intergenic regions, introns, upstream/downstream regions of genes and 3′/5′ prime UTRs) occupy the largest proportion (~ 99.09%) of the genomes, whereas the variants located in coding regions such as synonymous and missense only account for no more than 1% (Supplementary Table 4). The gene information mainly includes gene name, location, symbol, gene type and functional descriptions (Figure 3A). The breed information includes breed name, distribution, usage, and phenotypic characteristics (Figure 3B). The phenotype information consists of five categories such as production and reproduction, meat and carcass, milk, disease-resistance, and wool traits (Figure 3C).

**Table 1:** Data statistics of iSheep.

**Data content**	**Data statistics**
Variants	82,689,498
SNPs	70,370,968
INDELs	12,318,530
Genes	26,802
lincRNA	3,481
Protein coding	19,953
Pseudogene	2,949
misc RNA	255
tRNA	55
miRNA	106
Samples	
Whole-genome sequencing (WGS)	355
Ovine Illumina 50K SNP BeadChip	1,512
Ovine Infinium HD SNP BeadChip (600K)	911
Phenotypes	
Breeds	1,417
Associations	922
Ontology	110
Disease-resistance trait	23
Meat and carcass trait	33
Milk trait	7
Production and reproduction	33
Wool trait	14
Publications	52

### Retrieving and Browsing Data

iSheep provides an online documentation to help users familiarize with the database and a convenient way to retrieve and download data through a uniform user interface. The advanced search engines are designed in different modules to improve the usability and accessibility.

(*i*) In the Breeds module, basic information about 1,417 breeds is listed by integrating the content of 20 resources (Supplementary Table 2). Users are able to search breeds of interests through the key words such as breed name and/or country name, and then filter the breed by usage or body size. Detailed information including images and morphological, production, reproduction and other phenotypic characteristics has been curated and integrated in system, and is linked with breed name for further displayed.

(*ii*) In the GWAS module, 922 variant-trait associations and 111 traits are manually curated from 52 publications (Supplementary Table 3). To unify the representation of biological traits, the trait entities are divided into a suite of ontologies by using the standard of sheep QTLdb (Hu et al., 2019b). When a trait term is selected, basic descriptive information on association, trait and publication will be automatically mapped and displayed on the right panel (Figure 3C), where users could view the detailed information for different species. Additionally, for each publication, its bibliographic details are collectively summarized in the Publications module. Therefore, the mapping between GWAS traits and ontology terms would be useful to identify new potential genetic variants by providing all related associations across different species.

(*iii*) In the Variants module, variants called from 355 whole genome sequences and SNP BeadChip of 2,423 individuals, and annotated genes are showed. To support information search and exploration, powerful retrieve functions are designed for users to filter variants by name, position, consequence types and/or minor allele frequency, while users can also choose the sequencing technologies to locate the concerned data sets. Typically, the elaborate information of each variant marked with annotation label, for example functional change or not, makes the selection of the specific variation much easier. Through clicking variant, the detailed and structured information (e.g., genes, SNPs and INDELs) with a visualized bar is shown, and also, the concerned genotypes of this variant in 355 sheep WGS samples are showed (Figure 3D). Specially, if the variant maps to a SNP site in 600 K and/or 50 K BeadChip, the SNP genotypes in the chip(s) will be linked out, with the total number of the samples pooled in chips up to 2,423 individuals from 47 sheep breeds.

In other modules, such as the Samples and Genes, there are searching engines for further data query, providing the external links to the data sources to find more detail information.

### Morphological and Phenotypic Traits Survey Using iSheep

The integration and correlation of multiple data types in sheep make iSheep a knowledge base, which brings a convenient way for users. Based on the Variants module, users can obtain all corresponding information in other different modules (i.e., Genes, Breeds, Samples and GWAS) of iSheep. For example, through the Associations in GWAS module, users can filter out the traits “white to black” or “white spotted”, and then choose one variant (e.g., “oas24670730”) in the result page (Figure 3C). The detailed information of this variation will be listed and the extensional information of Beadchip data will be linked out (Figure 3D). In this example, the breed “Duolang” will be extracted because of its the highest mutation frequency among all breeds (Figure 3E). Besides that, the image and quantifiable phenotype data of this sheep breed (Figure 3B) and the related genes (Figure 3A) of this variant will be connected.

### Online Tools of iSheep

iSheep provides two online tools for users to analyze data. The first one is Comparison, which can be used to compare SNPs between two or more individuals. The results show the information of different samples' genotypes on variant sites and make it more convenient for users to focus on their interested SNPs. The second is Genome browse, which helps users to better visualize the locus of variants in the genome. Users can easily visualize the interested region with genes, transcripts, SNPs, INDELs and other elements. Further, users can export their results in the SVG format.

## Discussion

By integrating omics data of sheep, variants, and phenotype, we have developed a new sheep database, iSheep, which provides an easy access for users to download raw re-sequencing/variant data, perform their personalized analyses online, and visualize and export results. Compared with some available sheep databases, such as ISGC and dbSNP which are not updated in time, iSheep excels in the following aspects: (*i*) Fast updated with multiple data types and resources (i.e., WGS data, variant data, gene data, breed data and GWAS data). Besides the domestic sheep data, iSheep provides raw re-sequencing and variant data for various wild sheep including argali (*O. ammon*), Asian mouflon (*O. orientalis*), bighorn (*O. canadensis*) and thinhorn (*O. dalli*), which makes it more convenient to investigate demographic history and domestication of sheep; (*ii*) Multiple options for keyword searching; (*iii*) Comprehensive annotations for variants and phenotypes, comprising 922 variant-trait associations for 110 phenotypic traits; and (*iv*) A user-friendly website with functional online tools including Comparison and Genome Browse.

The overall goal of iSheep is to provide a comprehensive resource for sheep studies. In the future, we will continue to improve gene annotations and exploit additional applications after integrating new data types such as transcriptomics and proteomics, and continue to collect more phenotypic data to increase breed traceability. The development of online tools for omics data analysis will also be our focus. In addition, we are striving to develop an online tool for performing imputation of missing genotypes for the same SNP position based on existing genotypes in our database.

## Data Availability Statement

The original contributions presented in the study are included in the article/Supplementary Material, further inquiries can be directed to the corresponding author/s.

## Author Contributions

M-HL and W-MZ conducted and designed this study. Z-HW, Q-HZ, J-WZ, D-MT, H-LK, C-PL, and S-SZ collected data and implemented the database. XL, Z-HW, and Q-HZ wrote the first draft of the manuscript. M-HL and W-MZ reviewed and edited the final manuscript. All authors reviewed and approved the paper for publication.

## Conflict of Interest

The authors declare that the research was conducted in the absence of any commercial or financial relationships that could be construed as a potential conflict of interest. The reviewer FW declared a past co-authorship with several of the authors XL, M-HL to the handling Editor.

## Publisher's Note

All claims expressed in this article are solely those of the authors and do not necessarily represent those of their affiliated organizations, or those of the publisher, the editors and the reviewers. Any product that may be evaluated in this article, or claim that may be made by its manufacturer, is not guaranteed or endorsed by the publisher.
